# COVID-19 and the risk of CNS demyelinating diseases: A systematic review

**DOI:** 10.3389/fneur.2022.970383

**Published:** 2022-09-20

**Authors:** Itay Lotan, Shuhei Nishiyama, Giovanna S. Manzano, Melissa Lydston, Michael Levy

**Affiliations:** ^1^Division of Neuroimmunology and Neuroinfectious Disease, Department of Neurology, Massachusetts General Hospital and Harvard Medical School, Boston, MA, United States; ^2^Treadwell Virtual Library for the Massachusetts General Hospital, Boston, MA, United States

**Keywords:** COVID-19, multiple sclerosis (MS), neuromyelitis optica spectrum disorder (NMOSD), myelin oligodendrocyte glycoprotein antibody-associated disease (MOGAD), diagnosis, relapse, exacerbation

## Abstract

**Background:**

Viral infections are a proposed possible cause of inflammatory central nervous system (CNS) demyelinating diseases, including multiple sclerosis (MS), neuromyelitis optica spectrum disorder (NMOSD), and myelin oligodendrocyte glycoprotein antibody-associated disease (MOGAD). During the past 2 years, CNS demyelinating events associated with severe acute respiratory syndrome coronavirus 2 (SARS-CoV-2) infection have been reported, but causality is unclear.

**Objective:**

To investigate the relationship between CNS demyelinating disease development and exacerbation with antecedent and/or concurrent SARS-CoV-2 infection.

**Methods:**

A systematic literature review of all publications describing either a new diagnosis or relapse of CNS demyelinating diseases (MS, NMOSD, MOGAD) in association with SARS-CoV-2 infection was performed utilizing PRISMA guidelines. Descriptive statistics were used for data analysis, using a case analysis approach.

**Results:**

Sixty-seven articles met the inclusion criteria for the study. Most of the reported cases of NMOSD (*n* = 13, 72.2% of reported cases) and MOGAD (*n* = 27, 96.5% of reported cases) were of new disease onset, presenting with typical clinical and radiographic features of these conditions, respectively. In contrast, reported MS cases varied amongst newly diagnosed cases (*n* = 10, 10.5% of reported cases), relapses (*n* = 63, 66.4%) and pseudo-relapses (*n* = 22, 23.2%). The median duration between COVID-19 infection and demyelinating event onset was 11.5 days (range 0–90 days) in NMOSD, 6 days (range−7 to +45 days) in MOGAD, and 13.5 days (range−21 to +180 days) in MS. Most cases received high-dose corticosteroids with a good clinical outcome.

**Conclusion:**

Based upon available literature, the rate of CNS demyelinating events occurring in the setting of preceding or concurrent SARS-CoV-2 infection is relatively low considering the prevalence of SARS-CoV-2 infection. The clinical outcomes of new onset or relapsing MS, NMOSD, or MOGAD associated with antecedent or concurrent infection were mostly favorable. Larger prospective epidemiological studies are needed to better delineate the impact of COVID-19 on CNS demyelinating diseases.

## Introduction

Multiple sclerosis (MS), neuromyelitis optica spectrum disorder (NMOSD), and myelin oligodendrocyte glycoprotein antibody-associated disease (MOGAD) are immune-mediated inflammatory demyelinating diseases of the central nervous system (CNS). While the cause of these conditions is unknown, it is proposed that an interaction between genetic predisposition and behavioral, environmental, and personal factors contribute to disease development. Among the environmental factors involved, viral infections are considered a possible triggering factor.

Prior studies have shown a higher rate of multiple sclerosis (MS) exacerbation in temporal association with viral infections, especially upper respiratory tract infections caused by influenza A virus and Epstein Barr virus (EBV) (1). EBV has also been proposed as a causal agent in the onset of MS (2, 3). Likewise, preceding infections have been proposed as a possible trigger for the induction of pathogenic mechanisms leading to the development of NMOSD and MOGAD (4–10).

During the past 2 years, neurological complications associated with SARS-CoV-2 infection, the aetiologic agent of the coronavirus disease 2019 (COVID-19), have been reported. Some of these complications are thought to be caused by direct damage to the nervous system as a result of direct viral invasion (11). However, in most cases, the severe acute respiratory syndrome coronavirus 2 (SARS-CoV-2) CSF RNA test is negative, and an immune-mediated mechanism is postulated (12–15). In this latter category, reports of MS, NMOSD, and MOGAD cases presenting either as new diagnoses or disease relapses in temporal association with COVID-19 have been accumulating.

This systematic review aims to summarize the available data regarding the occurrence of new disease onset and disease exacerbation of MS, NMOSD, and MOGAD associated with SARS-CoV-2 infection.

## Materials and methods

This systematic literature review was performed utilizing PRISMA guidelines. Electronic searches for published literature were conducted by a medical librarian using Ovid MEDLINE (1946 to present), Embase.com (1947 to present), and Web of Science (1900 to present). The searches were run in December 2021. A search update was run in May 2022.

The search strategy incorporated controlled vocabulary and free-text synonyms for the concepts of multiple sclerosis (MS), neuromyelitis optica spectrum disorder (NMOSD), myelin oligodendrocyte glycoprotein antibody-associated disease (MOGAD), relapse, new diagnosis, and COVID-19. The full database search strategies are documented in Appendix 1. No restrictions on language or any other search filters were applied. All identified studies were combined and de-duplicated in a single reference manager (EndNote). The citations were then uploaded into Covidence systematic review software.

The full reference list of all selected papers was screened for additional relevant sources. Publications meeting the purpose of the review that were not identified through the initial electronic search were added manually to the final review. The paper selection and data extraction process were carried out independently by two authors (IL and SN), with a third author available in case of disagreements.

To ensure maximal coverage of the currently available data pertinent for the topic of this review, we included all available case reports, case series, and cohort studies that met the pre-defined case selection criteria, presented either as manuscripts in peer-reviewed scientific journals or as posters or oral presentations in a scientific congress.

Descriptive statistics was used to present the data from reported cases, using a case analysis approach. Cases with missing data points were excluded from the analysis of the missing variable.

### Case selection

We included patients of any age with confirmed COVID-19 and case description consistent with a new diagnosis or a relapse of MS, NMOSD, or MOGAD, in accordance with the 2017 revised McDonald criteria for MS (16), the 2015 international consensus diagnostic criteria for NMOSD (17), and the international recommendations on the diagnosis of MOGAD (18), respectively. Patients fulfilling a diagnosis of clinically isolated syndrome (CIS), considered as having a high likelihood of MS, were included as well. A relapse was defined as a clinical episode reflecting a focal or multifocal CNS demyelinating event lasting at least 24 h, in the absence of fever or active infection (16). When such an event was reported during an acute febrile state related to COVID-19, it was regarded as a pseudo-relapse, even when considered a relapse in the original publication.

COVID-19 cases were included if meeting one of the following criteria, as defined by the United States Centers for Disease Control and Prevention and the Infectious Diseases Society of America: (1) clinical symptoms consistent with COVID-19 without laboratory confirmation in the absence of an alternative explanation, (2) nasopharyngeal swab positive for COVID-19 PCR with or without symptoms, or (3) positive COVID-19 serologies with or without symptoms (19, 20).

No assumptions were made regarding the duration between COVID-19 and the onset of neurological manifestations. Missing data was noted as not available.

### Exclusion criteria

Cases describing clinical manifestations consistent with demyelinating events of the CNS (i.e., optic neuritis, transverse myelitis, acute disseminated encephalomyelitis, etc.) not fulfilling the diagnostic criteria for MS, NMOSD, or MOGAD as described above, were excluded from this review. Papers reporting a suspected diagnosis of COVID-19 that do not fulfill the diagnostic criteria described above, and papers not available for full-text review were also excluded.

## Results

**Figure 1:**
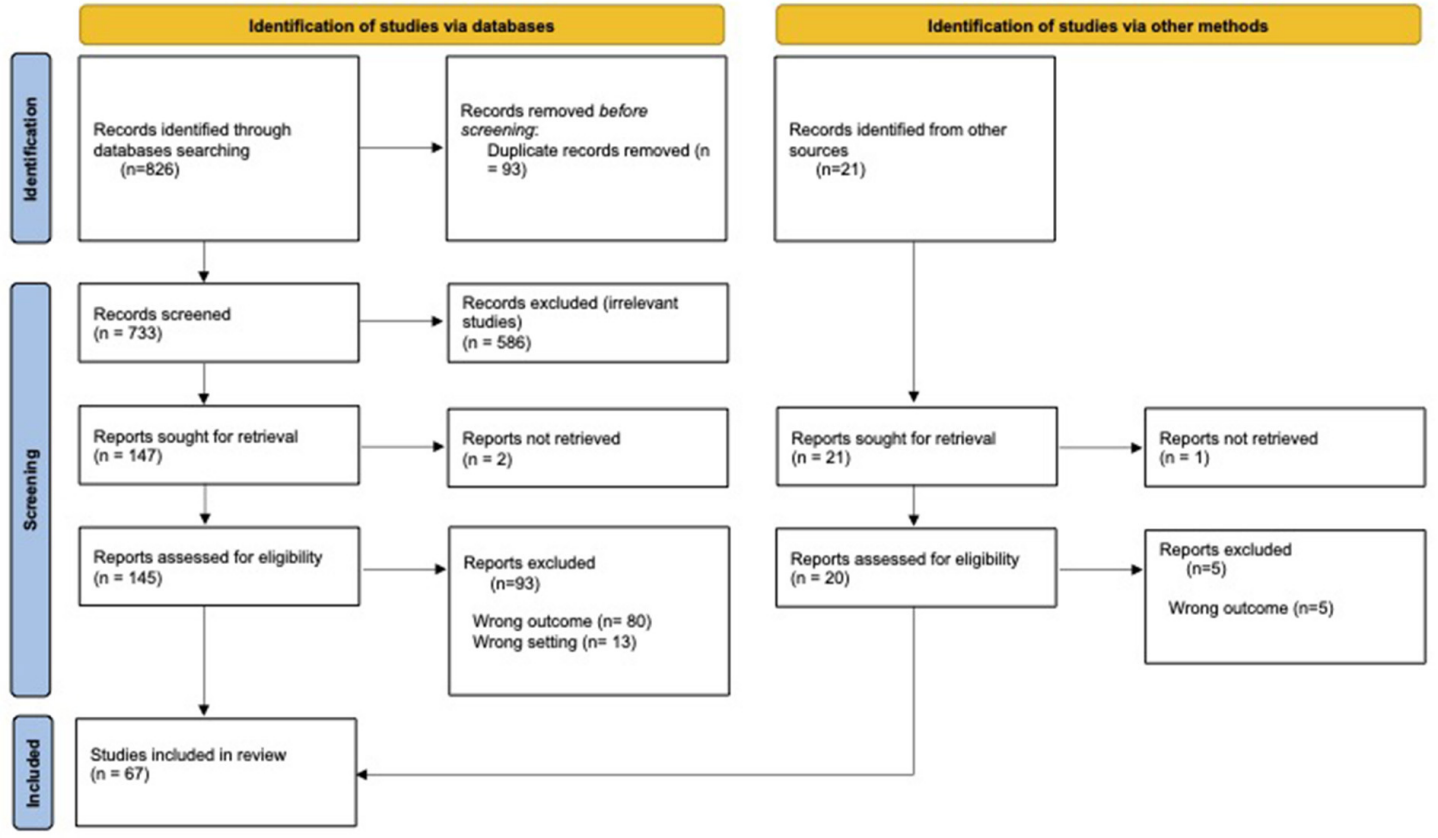
PRISMA flow chart of the article selection process.

Sixty-seven articles were included in the final review. Twelve articles describe post-COVID-19 NMOSD (21–32), 25 describe post-COVID-19 MOGAD (33–56), and 29 describe post-COVID-19 MS (57–85). One paper describes three patients with post-COVID-19 demyelinating events, of which one is NMOSD, one- MOGAD, and one- clinically isolated syndrome (CIS) (86). Another paper describes various CNS inflammatory diseases, of which three were MOGAD and one—NMOSD (87). A PRISMA flow chart illustrating the article selection process is presented in Figure 1.

### Post-COVID-19 NMOSD

Cases of post-COVID-19 NMOSD are summarized in Table 1.

**Table 1:** COVID-19 and NMOSD: Cases of para- and post-infectious disease development, relapse or pseudo-relapse.

**References**	**Number of patients**	**Diagnosis**	**Gender**	**Age**	**Ethnicity**	**Clinical presentation**	**AQP4-IgG status**	**Time from diagnosis of COVID-19 to clinical onset**	**Method of COVID-19 diagnosis**	**CSF SARS-CoV-2 PCR**	**Treatment of acute attack**	**Outcome**
Barone et al. (22)	1	New onset	M	35	NA	ON + acute myositis	Positive (titer not reported)	1 month	Clinical criteria + serology	NA	IVMP	Poor recovery of vision, full recovery of muscle symptoms
Batum et al. (23)	1	New onset	F	50	NA	LETM	Positive (titer not reported)	Concomitant	Clinical symptoms	NA	IVIG 0.4 g/kg for 5 days, then PLEX (10 courses every other day) + IVMP (750 mg every other day)	Some improvement in sensory function in the upper limbs, no motor improvement
Shaw et al. (29)	1	New onset	M	NA *Septuagenarian	NA	ON + TM	Positive (titer not reported)	9 days	SARS-CoV-2 PCR	NA	NA	Died due to sepsis and multiorgan failure
Chuang and Miskin (24)	1	New onset	NA	NA	NA	LETM + APS	Positive (titer not reported)	Neurological symptoms appeared shortly after COVID-19 diagnosis	Clinical symptoms + serology	NA	NA	NA
Corrêa et al. (25)	1	New onset	F	51	Caucasian	Encephalomyeloradiculitis	Positive (titer not reported)	2 weeks	SARS-CoV-2 PCR	Negative	IVMP 1 gr X5 days followed by PLEX	Remarkable improvement
Nasreldein et al. (28)	1	New onset	F	56	NA	BON+ diencephalic syndrome (lethargy and disorientation)	NA	2 weeks	SARS-CoV-2 PCR	NA	IVMP 1 gr/day (treatment duration not reported)	Deceased
Hooshmand et al. (27)	1	New diagnosis *Patient suffered from intractable emesis and visual loss 30 years prior	M	49	NA	ON	Positive (1:10 by FACS assay)	2 weeks	SARS-CoV-2 PCR	NA	NA	NA
Shukla et al. (30)	1	New onset	F	13	Asian	BON, APS, brainstem syndrome, cerebral syndrome	Negative	NA	Clinical criteria + serology	NA	CS, IVIG, Rituximab	Improved
Khair et al. (86)	1	New onset *Patient had an undiagnosed ADEM-like demyelinating episode 6 month prior	F	14	NA	Left eye blurring of vision, neck pain, generalized fatigue, and right leg numbness	Positive (titer not reported)	Concomitant	SARS-CoV-2 PCR	NA	NA	NA
Ghosh et al. (26)	1	New onset	M	20	Asian-Indian	APS + LETM	Positive (titer not reported)	5 days	SARS-CoV-2 PCR	NA	IVMP 1 gr/d for 5 days; RTX	Some improvement of the motor power in all limbs and resolution of the sensory symptoms
Jentzer et al. (31)	1	New onset	F	71	Caucasian	LETM	Positive (titer not reported)	3 months	SARS-CoV-2 PCR	NA	NA	NA
Das et al. (32)	1	New onset	F	16		ON + LETM	Negative	4 months	Clinical symptoms + serology	NA	IVMP + oral prednisone taper + RTX	Improvement of vision; outcome of myelopathic symptoms not reported
Aubart et al. (87)	1 *Also describes 3 MOGAD cases	New onset	F	14	NA	ON	Positive (titer not reported)	NA *Inclusion criteria required positive testing for SARS-CoV-2 infection performed <6 weeks before onset of neurological symptoms or seroconversion following the symptoms with a prior history of SARS-CoV-2 exposure.	SARS-CoV-2 PCR	NA	IVMP	Complete recovery
Apostolos-Pereira et al. (21)	34 NMOSD patients who developed COVID-19	Five patients (15%) presented neurologic manifestations (relapse or pseudo exacerbation) during or after SARS-CoV2 infection	NA	48, 25, 16, 22, 32	NA	2- ON, 1-visual acuity worsening in previous ON, 1-TM, 1- not reported	15 patients- positive; 7- negative; 7- not tested (all patients fulfilled the NMOSD diagnostic criteria). *The antibody status of the five patients who had relapse is not specified.	In one patient neurological symptoms appeared 7 days after the viral infection, in one- concomitantly with the febrile illness, in the other 3- not reported	18- SARS-CoV-2 PCR; 16- Clinical symptoms *The method of diagnosis of the five patients who had relapse is not specified.	NA	2- oral CS; 2- IVMP; 1- not reported	3- Good recovery; 1- Poor recovery; 1- Worsening of EDSS from 4.0 to 5.0

APS, area postrema syndrome; BON, bilateral optic neuritis; CS, corticosteroids; IVIG, intravenous immunoglobulins; IVMP, intravenous methylprednisolone; LETM, longitudinally extensive transverse myelitis; ON, optic neuritis; PLEX, plasma exchange; RTX, rituximab; TM, transverse myelitis.

Collectively, 13 case reports and one case series describe the occurrence of 18 NMOSD-related clinical events in the context of COVID-19 (21–32, 86, 87). Eight patients were females, four were males, and in six cases the patients' sex was not reported. The mean age was 33.24 ± 18.5 years.

Ten case reports describe the onset of newly diagnosed NMOSD in people without previous neurological disease (22–26, 28–30, 32, 87). Two case reports describe people with previously undiagnosed neurological disease who then presented with a second clinical manifestation in temporal association to COVID-19, leading to an NMOSD diagnosis (27, 86). In one case, the aquaporin-4 antibodies (AQP4 Abs) were retrospectively found to be positive in a stored serum sample drawn 11 months before SARS-CoV-2 infection and more than a year before the clinical onset of NMOSD (31). Apostolos-Pereira et al. report a series of 34 NMOSD patients who developed COVID-19. Five of these patients (15%) developed neurological manifestations that were regarded as relapse or pseudo-relapse during or after SARS-CoV2 infection (21).

In 10 case reports, AQP4-IgG Abs were positive (22–27, 29, 31, 86, 87). In two case reports the AQP4 abs were negative (30, 32) and each fulfilled the diagnostic criteria for seronegative NMOSD. In one report, AQ4 serostatus was not reported (28). In the case series by Apostolos-Pereira et al., 15 patients tested positive for the AQP4-IgG Abs, 7 tested negative, and in 7 the antibody testing was not available (all patients fulfilled the NMOSD diagnostic criteria). The AQP4 antibody status of the five patients who had a relapse was not specified (21).

Neurological symptoms appeared after a median of 11.5 days (range 0–90 days) from COVID-19 diagnosis. In all cases, COVID-19 symptoms preceded the occurrence of neurological symptoms.

Treatment consisted of corticosteroid (CS) monotherapy in seven cases (21, 22, 28, 87), CS + rituximab in two cases (26, 32), intravenous methylprednisolone (IVMP)+ intravenous immunoglobulin (IVIG)+plasma exchange (PLEX) in one case (23), CS +IVIG+ rituximab in one case (30), and CS + PLEX in one case (25). In the remaining six cases, the treatment regimen was not reported (21, 24, 27, 29, 86). A favorable outcome (i.e., improvement of neurological symptoms) was reported in eight patients (21, 25, 26, 30, 32, 87), while poor neurological outcome (i.e., worsening of neurological disability) was reported in four patients (21–23). Two patients deceased due to systemic complications (28, 29). The clinical outcome was not reported for the remaining four patients (24, 27, 31, 86).

### Post-COVID-19 MOGAD

Post-COVID-19 MOGAD cases are summarized in Table 2.

**Table 2:** COVID-19 and MOGAD: Cases of para- and post-infectious disease development or exacerbation.

**References**	**Number of patients**	**Diagnosis**	**Gender**	**Age**	**Ethnicity**	**Clinical presentation**	**Method of COVID-19 diagnosis**	**CSF SARS-CoV-2 PCR**	**Time from diagnosis of COVID-19 to clinical onset**	**Treatment**	**Outcome**
Zhou et al. (44)	1	New onset	M	26	Hispanic	BON +TM	SARS-CoV-2 PCR	Negative	A few days	IVMP 1 gr X 5d followed by oral prednisone taper	Rapid improvement in vision, outcome of myelopathic symptoms not reported
Ide et al. (35)	1	New onset	F	24	NA	ON+ TM (diagnosed as ADEM d/t additional brain lesions)	SARS-CoV-2 PCR	Negative	3 weeks	IVMP 1 gr X5 days followed by prednisolone taper	Visual symptoms Improved spontaneously; other symptoms improved after treatment
Khan et al. (36)	1	New onset	M	11	NA	BON	SARS-CoV-2 PCR	NA	4 days	IVMP + prednisone taper	Improved vision
Kogure et al. (37)	1	New onset	M	47	Asian	ON (clinically unilateral, but bilateral optic nerve enhancement on MRI)	SARS-CoV-2 PCR	Negative	Concomitant	IVMP 1 gr X 3 days + prednisone taper	Rapid improvement in pain and vision
Pinto et al. (40)	1	New onset	F	44	NA	CNS inflammatory vasculopathy	SARS-CoV-2 PCR	Negative (repeated twice)	7 days	IVMP 1 gr X5 days followed by oral prednisolone 60 mg/d, + PLEX	Rapid clinical improvement
Woodhall et al. (43)	1	Relapse	F	39	NA	ON	SARS-CoV-2 PCR	NA	6 days	IVMP 1 g/day for 5 days followed by five cycles of PLEX	Partial improvement
Sawalha et al. (41)	1	New onset	M	44	Hispanic	BON	SARS-CoV-2 PCR	NA	One week	IVMP 1 g/day for 5 days followed by prednisone taper	Complete recovery in one eye, remarkable recovery but not complete in the other eye
Khair et al. (86)	1 *Also describes one case of NMOSD and one CIS	New onset *Concomitant NMDAR Abs	F	16	NA	Headache, blurred vision, leg numbness, and weakness.	SARS-CoV-2 PCR	NA	Concomitant	NA	NA
Lindan et al. (38)	1	New onset	M	4	NA	Seizures, facial palsy, and four limb dysfunction	SARS-CoV-2 serology	NA	NA	IVMP	Marked improvement
Peters et al. (39)	1	New onset	M	23	NA	Headaches and dysesthesia followed by seizures, inattention and cognitive slowing	SARS-CoV-2 PCR	Negative	Initial neurological symptoms developed concomitantly with positive COVID-testing; further symptoms developed over the next 4 weeks	IVMP 1 gr X5 days followed by oral steroid taper	Gradual clinical and radiological resolution
Vraka et al. (42)	1 *Describes another case of encephalopathy with negative MOG-IgG	New onset	F	13 months	NA	ADEM	SARS-CoV-2 PCR	Negative	Concomitant	Steroids	Gradual improvement
Ahsan et al. (33)	1	New onset	F	7	NA	ADEM	SARS-CoV-2 serology	NA	Neurological symptoms preceded COVID-19 by a week	IVIG 2 g/kg over 3 days	Gradual improvement (almost returned to her baseline with mild dysarthria)
de Ruijter et al. (34)	1	New onset	M	15	Caucasian	BON	Clinical criteria	NA	A few weeks	IVMP 1 gr/d for 3 days	Improved (almost full recovery)
Durovic et al. (47)	1	New onset	M	22	NA	Encephalitis	SARS-CoV-2 PCR	Negative	3 days	IVMP 1 gr/d for 5 days	Complete clinical and radiological resolution
Jumah et al. (46)	1	New onset *Concomitant HHV6 infection	M	61	NA	LETM	SARS-CoV-2 PCR + serology	Negative	1 week	IVMP 1 gr/d for 5 days + prednisone taper + Gancyclovir, PLEX (7 sessions)	Marked improvement
Sinha et al. (45)	1	New onset	F	11	NA	BON	SARS-CoV-2 PCR	NA	3 days	IVMP 1 gr/d + IVIG 2gr/kg for 5 days + prednisone taper	Improved
Yang et al. (55)	1	New onset	M	57	NA	LETM	SARS-CoV-2 PCR	NA	3 weeks	IVIG × 4 days, then five sessions of PLEX, then IVMP 1 gr/d for 5 days + steroid taper	NA
Assavapongpaiboon et al. (50)	1	New onset	F	35	Thai	BON	SARS-CoV-2 PCR	Negative	1 week	IVMP 1 gr/d for 5 days + steroid taper	Improved
Dias da Costa et al. (52)	1	New onset	M	31	NA	LETM	SARS-CoV-2 serology	Negative	21 days	IVMP 1 gr/d for 5 days + steroid taper	Almost complete resolution of motor and sensory symptoms, mild urinary symptoms
Doukas et al. (53)	1	New onset	M	40	NA	TM	SARS-CoV-2 serology	NA	12 days	IVMP 1 gr/d for 5 days + steroid taper	Gradual improvement
Jossy et al. (54)	1	New onset	M	38	NA	ON	SARS-CoV-2 serology	NA	6 weeks	IVMP 1 gr/d for 3 days + steroid taper	Complete resolution
Rojas-Correa et al. (49)	1	New onset	M	69	NA	BON	Clinical criteria	Negative	45 days	IVMP 1 gr/d for 5 days + steroid taper	Improved
Sardar et al. (48)	1	New onset	F	38	NA	BON *Diagnosed with concomitant idiopathic intracranial hypertension	Clinical criteria	NA	2 weeks	IVMP for 5 days, PLEX, IVIG for 5 days Acetazolamide	Significant improvement
Žorić et al. (51)	1	New onset	M	63	NA	ON	SARS-CoV-2 serology	NA	4 weeks	IVMP 1 gr/d for 5 days + steroid taper	Improved
Aubart et al. (87)	3 *Also describes one case of NMOSD	New onset	2M, 1F	1.5, 4, 10	NA	ADEM	SARS-CoV-2 PCR or serology	NA	NA *Inclusion criteria required positive testing for SARS-CoV-2 infection performed <6 weeks before onset of neurological symptoms or seroconversion following the symptoms with a prior history of SARS-CoV-2 exposure.	2- IVMP, 1- not treated	Complete recovery
Cay-Martínez et al. (56)	1	New onset	F	7	NA	ADEM	SARS-CoV-2 serology	Negative	1 week	IVMP + PLEX + IVIG + oral prednisone taper	Resolution of facial and upper extremity weakness, mild improvement in leg weakness

ON, optic neuritis; BON, bilateral optic neuritis; TM, transverse myelitis; LETM, longitudinally extensive transverse myelitis; ADEM, acute demyelinating encephalomyelitis; IVMP, intravenous methylprednisolone; IVIG, intravenous immunoglobulins; PLEX, plasma exchange.

A total of 28 cases of MOGAD occurring in temporal relation to COVID-19 have been described (33–47, 56, 86, 87). Seventeen were males, and 11 were females. The mean age was 28.1 ± 20.3 years (range 1–69 years; 10 patients <18 years old). The median time between COVID-19 and neurological symptoms was 6 days (range−7-+45 days). In one case, the neurological symptoms preceded the diagnosis of COVID-19 by 1 week (33). In four cases, neurological symptoms developed concomitantly with COVID-19 (37, 39, 42, 86), and in the remaining 23, COVID-19 diagnosis preceded the onset of neurological symptoms. In 27 (96.5%), a new diagnosis of MOGAD was made in people without prior neurological disease. In one case, a relapse occurred in a patient with known MOGAD (43). The MOG-IgG antibodies were positive in all cases. In one case, NMDAR antibodies and MOG-IgG antibodies were detected concomitantly (86). In another case, human herpes virus 6 (HHV6) PCR was also positive (46). Eighteen patients were treated with CS alone (intravenous methylprednisolone followed by oral prednisone taper, *n* = 15; IVMP alone, *n* = 2; details of steroid regimen were not described, *n* = 1) (42). One patient was treated with intravenous immunoglobulins (IVIG) alone (33). One patient received IVMP+ IVIG (45), three received IVMP + PLEX (40, 43, 46), and three received IVMP+ PLEX+ IVIG (48, 55, 56). One patient was not treated (87). The treatment regimen was not described for one patient (38). Clinical improvement was reported for 26 patients (93%).

### Post COVID-19 MS

Table 3 illustrates MS cases occurring in the context of COVID-19.

**Table 3:** COVID-19 and MS: Cases of para- and post-infectious disease development, relapse or pseudo-relapse.

**References**	**Number of patients**	**Diagnosis**	**Gender**	**Age**	**Ethnicity**	**Clinical presentation**	**Method of COVID-19 diagnosis**	**DMT before COVID-19 infection**	**Time from diagnosis of COVID-19 to clinical onset**	**CSF SARS-CoV-2 qPCR**	**Treatment**	**Outcome**
**Case reports**												
Moore et al. (77)	1	New onset	M	28	NA	Brainstem syndrome (vertigo, oscillopsia, diplopia, facial numbness)	Clinical criteria	None	Neurological symptoms appeared 10 days after COVID-19 symptoms	Negative	IVMP 1 g/day for 3 days followed by prednisone taper	Improved
Pignolo et al. (79)	2	1 new onset, 1 relapse	M, F	21,52	NA	Hand paresthesia and facial nerve palsy; Right-sided weakness and clumsiness	Clinical criteria, serology	None (new onset) Cladribine (relapse)	MS onset a few days after COVID-19; MS relapse 2 months after COVID-19	Negative in one case (new onset), NA in the other	IVMP 1 g/day for 5 days	Relapse- fully resolved, new onset disease- partial recover
Fragoso et al. (66)	1	New onset	F	27	Caucasian	Left side dysesthesia	Clinical criteria	None	6 months	Negative	NA	NA
Wildemann et al. (81)	1	MS relapse and Takotsubo cardiomyopathy	F	39	NA	Brainstem syndrome (dizziness, diplopia, dysarthria, dysphagia)	SARS-CoV-2 PCR	DMF	10 days	Negative	IVMP 2 gr/day for 5 days + PLEX (seven courses)	Slow improvement
Yavari et al. (82)	1	New onset	F	24	NA	Diplopia, facial nerve palsy, fingertips paresthesia	SARS-CoV-2 PCR	None	1 month	NA	IVMP 1 gr/day for 4 days	Improved
Palao et al. (78)	1	New onset	F	29	NA	ON	SARS-CoV-2 serology	None	2–3 weeks	Negative	IVMP 1 gr/day (treatment duration not reported) followed by oral prednisolone taper	Improved
Florae et al. (65)	1	Relapse *3 weeks post-partum	F	40	Caucasian	Right sided paresthesia and motor disability	SARS-CoV-2 PCR	None	No systemic symptoms, tested positive on swab PCR upon admission	NA	IVMP 1 gr/d for 3 days; hydroxychloroquine 4 g/day, lopinavir/ritonavir 4 tablets/day for 10 days, and azithromycin 1 g/day, for 3 days	Remission of neurological deficit after 2 weeks
Khair et al. (86)	1	CIS	M	8	NA	Double vision, worsening fine motor skills, and ataxic gait	Clinical criteria	None	1 month	NA	NA	NA
Kataria et al. (69)	3	Pseudo-relapse	2M, 1F	65, 52, 69	NA	Fatigue, general weakness	SARS-CoV-2 PCR	GA	Concomitant	NA	Only COVID-19 management	Improved to baseline status
Barzegar et al. (57)	1	Relapse	F	42	NA	Muscle aches, gait difficulty, sensory disturbances, and weakness on the right side	SARS-CoV-2 PCR	Fingolimod	Neurological symptoms preceded COVID-19 symptoms by 6 days	NA	Initially IVMP 1 gr/d for 3 days; then azithromycin, ceftriaxone, hydroxychloroquine, oseltamivir, and piperacillin/tazobactam	Gradual improvement
Domingues et al. (63)	1	CIS	F	42	NA	Left side paresthesia	Clinical criteria	None	Concomitant	Positive	No steroids, COVID-19 management not detailed	Full recovery
Jaisankar et al. (67)	1	Pseudo-relapse	M	45	Caucasian	Dysphagia, altered mental status, general deterioration	SARS-CoV-2 PCR	None	COVID-19 diagnosed 2 weeks prior to neurological deterioration. *Also diagnosed with acute renal failure, anemia, PE and sepsis.	NA	IVMP (dose and duration not reported). Received fluids, packed red blood cells and transfusions, anticoagulants, ciprofloxacin	Ongoing disability
Karsidag et al. (68)	2	Two patients with new-onset MS (^*^+1 ADEM)	1F, 1M	42, 32	NA	Jaw and left facial pain and paresthesia; numbness in left jaw	Clinical criteria	None	2–3 weeks; 4 months	1 Negative, 1 Positive	1-IVMP 1 gr/d for 7 days; 1- IVMP 1 gr/d for 10 days	Improved
Möhn et al. (76)	1	Relapse	M	42	NA	Gait and limb ataxia	SARS-CoV-2 PCR	Teriflunomide	Neurological symptoms preceded COVID-19 by 3 weeks	NA	IVMP 1 gr/d for 4 days	Initial improvement, then worsened concomitantly to COVID symptoms
Finsterer (84)	1	Relapse	F	27	NA	TM	Clinical criteria	IFNβ-1a	2 weeks	NA	CS	Slow improvement
**Observational case series and cohort studies of MS patients**												
Khurana et al. (71)	5 RRMS patients	1 relapse	3F, 2M	Mean (SD) age 35.60 (13.94)	NA	NA	SARS-CoV-2 PCR	Treated with DMT, type not specified	NA	NA	NA	NA
Maghzi et al. (73)	3 RRMS, 1 SPMS, 1 RIS	No relapses	3M, 2F	Mean 53.6	NA	NA	SARS-CoV-2 PCR	Teriflunomide	NA	NA	NA	NA
Mantero et al. (74)	7 RRMS patients	No relapses. 1 pseudo-relapse	5F, 2M	Mean 35.9 ± 11.4	NA	Left hand paresthesia	Clinical criteria	DMF	Concomitant	NA	NA	NA
Conway et al. (60)	72 RRMS, 21 SPMS, 8 PPMS, 2 CIS, eight related disorders	2/111 (1.8%) relapses, 19 (17.2%) pseudo-relapses and 27 (24.3%) with worsening of pre-existing MS symptoms. Five patients (4.5%) had new MRI lesions on T2 or T1Gd scans	85 females (77%)	Mean age 49 (SD 12.2) years	NA	NA	Clinical criteria	NA	NA		NA	NA
Chyzhyk et al. (59)	17 relapsing MS patients	No clinical or radiological signs of MS disease activity During 6 months of observation	4M, 13 F	Mean age 38 ± 7.6 years	NA	NA	Clinical criteria	Treated with DMT, type not specified	NA		NA	NA
Czarnowska et al. (61)	426 individuals with MS	27 patients (6.34%) had a relapse at 3 months after the initial infection	142M, 284F	Mean 40.27 ± 10.12	NA	Symptoms during the relapse were as following: pyramidal track symptoms (16 people), cerebellar symptoms (eight people), sensory deficit (four people), brainstem symptoms (3 people), urinary incontinence (1 person)	SARS-CoV-2 PCR (*n* = 361), SARS-CoV-2 serology (*n* = 24) or combination of tests	Interferon beta (*n* = 77); GA (*n* = 43); DMF (*n* = 171); teriflunomide (*n* = 34); fingolimod (*n* = 16); natalizumab (*n* = 29); ocrelizumab (*n* = 29); cladribine (*n* = 7); alemtuzumab (*n* = 1); mitoxantrone (*n* = 1); ozanimod (n=12); other (*n* = 12); none (*n* = 4) *Type of DMT in patients who relapsed not specified	The mean time for relapse occurrence after the SARS-CoV-2 infection was 43 days		All treated with IVMP 3–5 gr	NA
Michelena et al. (75)	41 MS patients with confirmed COVID-19 diagnosis	25 patients (61%) reported neurological worsening, three patients (7.7%) met criteria for relapse	24 F, 17M	Mean 42.9 years (SD 11.3)	NA	Motor (*n* = 12) Sensory (*n* = 10) Visual (*n* = 7) Balance disorders (*n* = 3) Memory (*n* = 6) Fatigue (*n* = 13)	SARS-CoV-2 PCR	35 treated with DMTs (23-oral DMTs, 4-injectables, 8-monoclonal antibodies)	Concomitant (*n* = 16), within the 1st month (*n* = 5), beyond the 1st month (*n* = 4)	NA	CS (type, dose, and duration not reported)	NA
Luetic et al. (72)	17 RRMS and 1 RIS patients	No MS relapses occurred during or after COVID-19 course.	13 F, 5M	Mean 41.2 ± 12.6	NA	NA	11- SARS-CoV-2 PCR; 8- Clinical criteria	Teriflunomide	NA	NA	NA	NA
Etemadifar et al. (64)	A retrospective cohort study comparing the risk of relapse in RRMS patients with (*n* = 56) and without COVID-19 (*n* = 69) *Within 6 months from COVID	4 patients in the MS-COVID-19 group (7.14%) had a relapse compared to 18 patients in the RRMS without COVID-19 group (26.09%). Incidence rate ratio: 0.275; *p* = 0.026	COVID-19 group: 40 F/15 M; non COVID-19 group: 62 F/ 7 M	COVID-19 group: 36.89 (±9.06); non-COVID-19 group: 36.19 (±8.97)	NA	2-limb paresthesia, 1-diplopia, 1-lower extremity weakness	SARS-CoV-2 PCR	Teriflunomide (*n* = 3); fingolimod (*n* = 9); DMF (*n* = 22); AZA (*n* = 5); Interferon ß 1b (*n* = 3; Interferon ß 1a (*n* = 6); GA(*n* = 3); RTX (*n* = 3); NTZ (*n* = 2) *Type of DMT in patients who relapsed not specified	Only reported that the 4 relapses in COVID-19 confirmed patients occurred after COVID-19 diagnosis	NA	NA	NA
Etemadifar et al. (83)	A prospective-retrospective hybrid single center cohort study comparing the risk of relapse during 1 year pre- and post-COVID-19 period in 53 RRMS patients *Some patients may have been included in the previous study by the same first author	11 patients (20.75%) in the post-COVID-19 period and 16 patients (30.19%) in the pre-COVID-19 period experienced a relapse (*p* = 0.30)	45 F, 8M	Mean 38.42 (SD 8.77)	NA	NA	Clinical criteria or SARS-CoV-2 PCR *Number of patients in each group not specified	IFN beta (*n* = 4); DMF (*n* = 21); teriflunomide (*n* = 1); GA (*n* = 1); fingolimod (*n* = 12); RTX (*n* = 9); AZA (*n* = 2); none (*n* = 3)	NA	NA	NA	NA
Barzegar et al. (58)	A retrospective observational study comparing the relapse rate among 41 MS patients with confirmed COVID-19 during a pre-defined at-risk period (from 2 weeks before to 5 weeks after COVID-19) and the previous 2 years	Five patients had a relapse during the defined at-risk period. Other two patients had neurological worsening that did not meet clinical relapse definition. Increased relapse rate during the at-risk period (RR: 2.566, 95% CI: 1.075–6.124, *P* = 0.034)	31 females, 10 males	Mean 35.10 ± 9.20	NA	NA	SARS-CoV-2 PCR	NA	All relapses occurred after the onset of COVID-19 (Mean 3.2 weeks, range 1–5 weeks)		NA	NA
Paybast et al. (85)	202 MS patients followed for 1 year	25 patients developed COVID-19, of which 1 (4%) had a relapse	164F, 37M	38.09 ± 10.44	NA	TM	SARS-CoV-2 PCR	NA	NA	NA	PLEX	NA
**General observational studies**												
Sandoval et al. (80)	13 pediatric patients with confirmed COVID-19 and new-onset neurological manifestations	1 patient with new-onset multifocal demyelination consistent with MS	M	14		ON, sixth nerve palsy, asymmetric paraparesis	SARS-CoV-2 PCR	None	No systemic symptoms, tested positive on swab PCR upon admission	NA	IVMP (dose and duration not reported)	Significant clinical improvement)
Khedr et al. (70)	439 patients with confirmed/probable COVID-19	2 MS relapse (among those with probable COVID-19, *n* = 62)	NA	NA	NA	NA	SARS-CoV-2 PCR	NA	NA	NA	NA	NA
Dhillon et al. (62)	Case series of 29 inpatients presented with COVID-19 and neurological disorders, 2 MS patients	1 MS relapse	M	56	White	Worsening of limb weakness and dysarthria	SARS-CoV-2 PCR	NA	NA	NA	NA	Ongoing disability

CIS, clinically isolated syndrome; DMT, disease modifying therapy; ON, optic neuritis; TM, transverse myelitis; IVMP, intravenous methylprednisolone; CS, corticosteroids; PLEX, plasma exchange.

Fifteen case reports and case series reported the occurrence of MS relapse/pseudo-relapse or the onset of a first demyelinating event consistent with MS or CIS in 19 patients (57, 63, 65–69, 76–79, 81, 82, 84, 86). Twelve observational case series and cohort studies documented the occurrence of relapses or pseudo-relapses among patients with a known diagnosis of MS and confirmed diagnosis of COVID-19 (58–61, 64, 71–75, 83, 85). Collectively, 54 relapses and 20 pseudo-relapses were reported in 804 patients (6.7 and 2.5%, respectively).

Three observational cohort studies of COVID-19 patients with various neurological manifestations reported MS cases (62, 70, 80). Overall, one case of multifocal demyelination consistent with MS and three MS relapses were reported among 481 patients (0.8%).

Considering all the reported cases, a total of 73 demyelinating events consistent with CIS/MS (10 new diagnoses and 63 relapses) and 22 events defined as pseudo-relapse were reported in 1,305 people (5.6 and 1.7%, respectively). Of these 73 events, 11 were in females, 10 in males, and sex was not reported in the remaining 64 cases. The mean age was 38.45 ± 15.93 years. Most relapses or first demyelinating events consistent with MS/CIS occurred after the onset of COVID-19. However, in two cases neurological symptoms preceded the diagnosis of COVID-19 by 6 and 21 days, respectively (57, 76). The median time from COVID-19 diagnosis to demyelinating event onset was 13.5 days (range−21-180 days).

Nine hundred eighty-six people with a known MS and COVID-19 diagnosis were reported. Of these, 624 (63.3%) were treated with various disease-modifying treatments (DMTs), 14 (1.5%) were not treated, and for 165 (16.7%), the information on DMTs was not reported. Twenty-one MS patients treated with DMTs (3.4%) had a relapse in temporal association with COVID-19. Five MS patients treated with DMTs (0.8%) had a pseudo relapse, and 144 (23.1%) did not have neurological worsening. The remaining 455 MS patients treated with DMTs (73%) were reported in larger cohorts in which some people were not treated. The information regarding relapses in these cohorts was not stratified between treated and untreated patients (61, 75).

Most MS/CIS cases received treatment with IVMP 1 gram for 3–5 days (57, 61, 65, 68, 76, 77, 79, 80, 82) and had a favorable outcome (57, 65, 68, 77–82). Treatment of pseudo-relapses was primarily focused on COVID-19 management, with return to baseline neurological status upon infection recovery (63, 69).

## Discussion

This systematic review summarizes the currently available data on the occurrence of demyelinating CNS events in the context of COVID-19. As noted, the vast majority of NMOSD and MOGAD cases represent newly diagnosed cases presenting with the typical clinical, radiological, and laboratory findings associated with these two disorders. In contrast, the MS cases described vary between newly diagnosed cases, relapses, and pseudo-relapses. The patients' age of diagnosis in the three disease groups was relatively similar to the age of diagnosis reported in the literature for non-COVID-19 related cases. The clinical presentations and treatment approach were also similar to non-COVID-19 related cases (for further details, please see Tables 1–3).

Several mechanisms involved in the pathogenesis of demyelinating events in the context of SARS-CoV-2 infection have been proposed. These may be related to either direct viral neurotropism or induction of aberrant immune response. The neurotrophic features of the Coronavirus family have been previously reported for the Middle East respiratory syndrome coronavirus (MERS-COV) and SARS-COV-1, and similar evidence has been occasionally reported for SARS-CoV-2 (88–91). However, the fact that the SARS-CoV-2 PCR test in the CSF was negative in many of the reported cases (25, 35, 37, 39, 40, 42, 44, 66, 68, 77–79, 81) would argue against this mechanism of direct pathogenicity. Conversely, some evidence favors the theory of para-infectious or post-infectious immune-mediated etiology. In fact, SARS-CoV-2 infection leads to hyperactivation of pro-inflammatory T cells resulting in increased levels of inflammatory cytokines and chemokines (92) and decreased regulatory T cells to impair immune response (93). The resulting pro-inflammatory hyperimmune state may activate specific immune-mediated mechanisms resulting in CNS inflammation and damage. The favorable response to immunotherapy in the majority of the reported cases appears to support this theory.

The distinction between relapse and pseudo-relapse may not always be straightforward. According to the 2017 McDonald criteria, a relapse should be defined in the absence of fever or acute infection; hence, new or worsening neurological symptoms developed during a febrile illness or in the presence of acute infection in a patient with a known diagnosis of MS should not be defined as true relapse, but rather regarded as a pseudo-relapse. However, there may be situations where the diagnosis of true relapse should still be considered even in the context of acute infection. For example, a true relapse should be considered if the onset of new symptoms is associated with clinical signs that can be attributed to a specific anatomical localization that has not been previously described or correlated with the presence of a new symptomatic MRI lesion. Following this rationale, a few of the described clinical worsening in MS cases were felt to be better classified as pseudo-relapses (67, 69).

Prior studies propose that MS relapses in temporal association with viral infections occur between 1 and 2 weeks before infection to 3–5 weeks after (94–98). Andersen et al. and Correale et al. reported that the highest frequency of relapses and infection-related MS attacks occurred during the first 2 weeks after infection onset (94). In the series reported by Sibley et al., the median time between the onset of infection and occurrence of MS exacerbation was 8 days (95). Buljevac et al. reported a mean duration of 9.5 days between the onset of infection and clinical MS exacerbation. Both Buljevac et al. and Correale et al. also compared the relapse rate ratio during different time intervals and found that the highest rate ratio was observed from weeks 1 to 4, while the exacerbation rate ratio for weeks 3–5 was lower and not statistically significant compared to the non-at risk period (97, 98). Considering these data, MS relapses occurring more than 4–5 weeks from an infection are probably not related to the prior infectious insult. Therefore, MS cases that occurred >6 weeks from COVID-19 (64, 66, 68, 79, 83), although included in this review in order to provide a comprehensive review of available data, are thought to be more likely coincidental and not related to the preceding infectious insult. Likewise, the relation between SARS-CoV-2 infection and the NMOSD and MOGAD cases developing >6 weeks after the infection (31, 32, 49, 54) remains uncertain. The case of MOGAD occurring in temporal association to both HHV6 and COVID-19 infection (46) may also confound the association between COVID-19 and MOGAD.

The use of disease-modifying therapies (DMTs) may be associated with an increased risk of viral and bacterial infections. Early in the course of the COVID-19 pandemic, this notion led to significant concerns regarding COVID-19 outcomes for people with neuroimmunological diseases. While some reports described a less favorable COVID-19 course in people treated with B-cell depleting agents, the use of other DMTs does not seem to be associated with such an increased risk (99–101). Another aspect of interest is whether the efficacy of DMTs is maintained during the pandemic. However, the currently available data is not sufficient to answer this question. While relapses were reported in only 3.4% of MS patients treated with DMTs, information about DMTs use and relapses was available for a relatively small proportion of patients (169/624, 27.1%). The fact that the majority of NMOSD and MOGAD cases reported are of newly diagnoses rather than relapses of previously diagnosed disease, may suggest that the efficacy of immunotherapy during the pandemic is maintained. In the series reported by Apostolos-Pereira et al., 97% of NMOSD patients (33/34) continued their prescribed immunotherapy during the pandemic. The relatively low incidence of neurological exacerbation reported by the authors (5/34, 15%) may further support this theory (21). Still, prospective studies comparing the rate of relapse between COVID-19 patients treated with DMTs and untreated patients are required to answer this question.

The current literature pertaining to the occurrence of demyelinating events in temporal association with COVID-19 is primarily composed of case reports, case series, and relatively small cohort studies. Therefore, while the rate of such events appears low based upon this review, especially considering the high prevalence of SARS-CoV-2 infection, the available data does not permit the determination of whether the rate of CNS demyelinating events (either new onset or true relapse) differs among people with confirmed COVID-19 compared to those who do not contract the infection. Additional questions that remain unanswered at this point are whether there are differences in the severity of demyelinating attacks and the response to acute treatments between demyelinating events occurring in association with COVID-19 and those not associated with the infection.

In conclusion, the rate of CNS demyelinating events occurring in the context of SARS-CoV-2 infection is relatively low given the global prevalence of infection. The clinical outcomes of new-onset or relapsing MS, NMOSD, or MOGAD associated with antecedent or concurrent SARS-CoV-2 infection is mostly favorable. Larger prospective epidemiological studies are needed to better characterize the impact of COVID-19 on CNS demyelinating diseases.

## Data availability statement

The original contributions presented in the study are included in the article/Supplementary material, further inquiries can be directed to the corresponding author.

## Author contributions

IL, SN, GM, and MiL contributed to the conception and design of the study. IL and MiL organized the database. IL and SN performed the data collection. IL wrote the first draft of the manuscript. MeL conducted the literature search. All authors contributed to manuscript revision, read, and approved the submitted version.

## Conflict of interest

The authors declare that the research was conducted in the absence of any commercial or financial relationships that could be construed as a potential conflict of interest.

## Publisher's note

All claims expressed in this article are solely those of the authors and do not necessarily represent those of their affiliated organizations, or those of the publisher, the editors and the reviewers. Any product that may be evaluated in this article, or claim that may be made by its manufacturer, is not guaranteed or endorsed by the publisher.
