# Supplementary material for: COVID-19 and the risk of CNS demyelinating diseases: A systematic review
Source: Front Neurol. 2022 Sep 20;13:970383. doi: 10.3389/fneur.2022.970383 (PMC9530047; doi:10.3389/fneur.2022.970383)
Supplement: Supplementary file 1 [file Data_Sheet_1.docx]

Appendix 1

**Supplemental Table 1, Full Search Strategy for MEDLINE Searched on 12/2/21**

| #1 | Multiple sclerosis.ti,ab,kw or (myelin oligodendrocyte glycoprotein antibody adj2 disease$).ti,ab,kw or NMOSD.ti,ab,kw or MOGAD.ti,ab,kw or neuromyelitis optica spectrum disorder$.ti,ab,kw or NMO Spectrum Disorder$.ti,ab,kw or Devic* Neuromyelitis Optica$.ti,ab,kw or Devic* Disease$.ti,ab,kw or Devic* Syndrome$.ti,ab,kw or exp Neuromyelitis Optica/ or exp Multiple Sclerosis/ or MS.ti or RRMS.ti,ab,kw or PPMS.ti,ab,kw or relapsing remitting MS.ti,ab,kw or remitting relapsing MS.ti,ab,kw or primary progressive MS.ti,ab,kw or acute relapsing MS.ti,ab,kw or Disseminated Sclerosis.ti,ab,kw or Acute Fulminating MS.ti,ab,kw |
| --- | --- |
| #2 | ((exp Coronavirus/ or Coronavirus Infections/ or pneumonia virus*.ti,ab,kf. or cov.ti,ab,kf.) and ((outbreak or wuhan).ti,ab,kf. or novel.af. or '19'.ti,ab,kf. or '2019'.ti,ab,kf. or epidem*.af. or epidemy.af. or epidemic*.af. or pandem*.af. or new.ti,ab,kf.)) or (coronavirus* or 'corona virus*' or ncov or '2019ncov' or 'covid*' or "sars cov 2" or 'sars2' or sarscov2 or sarscov-2 or "ncov 2019" or "sars coronavirus 2" or "sars corona virus 2" or "severe acute respiratory syndrome cov 2" or "severe acute respiratory syndrome cov2" or "severe acute respiratory syndrome cov*").ti,ab,kf. |
| #3 | (diagnos* or relaps* or exacerbat* or manifest* or recurrence$ or worsen* or flare up$ or flaring up or flareup$ or flare-up$ or symptom flare$ or symptom$ increase$ or symptom magnification$ or symptom exaggeration$ or disease burden).ti,ab,kw. or exp Recurrence/ or exp Symptom Assessment/ or Bystander Effect/ or di.fs or Diagnosis/ |
| #4 | 1 and 2 and 3 |
| #5 | limit 4 to dt="20191201-20220101" |

**Supplemental Table 2, Full Search Strategy for Embase.com Searched on 12/2/21**

| #1 | 'Multiple sclerosis':ti,ab,kw OR 'myelin oligodendrocyte glycoprotein antibody associated disease$':ti,ab,kw OR 'NMOSD':ti,ab,kw OR 'MOGAD':ti,ab,kw OR 'neuromyelitis optica spectrum disorder$':ti,ab,kw OR 'NMO spectrum disorder$':ti,ab,kw OR 'Devic* neuromyelitis optica$':ti,ab,kw OR 'Devic* disease$':ti,ab,kw OR 'Devic* syndrome$':ti,ab,kw OR 'myelooptic neuropathy'/exp OR 'Multiple Sclerosis'/exp OR 'myelin oligodendrocyte glycoprotein antibody associated disease'/exp OR MS:ti OR 'RRMS':ti,ab,kw OR 'PPMS':ti,ab,kw OR 'relapsing remitting MS':ti,ab,kw OR 'remitting relapsing MS':ti,ab,kw OR 'primary progressive MS':ti,ab,kw OR 'acute relapsing MS':ti,ab,kw OR 'Disseminated Sclerosis':ti,ab,kw OR 'Acute Fulminating MS':ti,ab,kw |
| --- | --- |
| #2 | ('coronavirus disease 2019'/exp OR 'covid-19 testing'/exp OR 'sars-cov-2 convalescent plasma'/exp OR 'coronavirus disease 2019 breathalyzer'/exp OR covid19:ti,ab,kw OR 'covid 19':ti,ab,kw OR 'sars-cov-2 vaccine'/exp OR 'sars-cov-2 antibody'/exp OR 'severe acute respiratory syndrome coronavirus 2'/exp OR 'sars coronavirus test kit'/exp OR 'sars cov 2':ti,ab,kw OR sars2:ti,ab,kw OR 'ncov 2019':ti,ab,kw OR 'sars coronavirus 2':ti,ab,kw OR 'sars corona virus 2':ti,ab,kw OR 'severe acute respiratory syndrome cov 2':ti,ab,kw OR 'severe acute respiratory syndrome cov2':ti,ab,kw OR 'coronavirinae'/exp OR 'coronavirus infection'/de OR coronavirus*:ti,ab,kw OR 'corona virus*':ti,ab,kw OR 'pneumonia virus*':ti,ab,kw OR cov:ti,ab,kw OR ncov:ti,ab,kw OR wuhan:ti,ab,kw) |
| #3 | 'diagnos*':ti,ab,kw OR 'relaps*':ti,ab,kw OR 'exacerbat*':ti,ab,kw OR 'manifest*':ti,ab,kw OR 'recurrence$':ti,ab,kw OR 'worsen*':ti,ab,kw OR 'flare up$':ti,ab,kw OR 'flaring up':ti,ab,kw OR 'flareup$':ti,ab,kw OR 'flare-up$':ti,ab,kw OR 'symptom flare$':ti,ab,kw OR 'symptom$ increase$':ti,ab,kw OR 'disease burden':ti,ab,kw OR 'symptom magnification$':ti,ab,kw OR 'symptom exaggeration$':ti,ab,kw OR 'Relapse'/exp OR 'diagnosis'/de OR 'symptom assessment'/exp OR 'bystander effect (cell)'/exp |
| #4 | #1 AND #2 AND #3 AND [2019-2030]/py |

**Supplemental Table 3, Full Search Strategy for Web of Science Searched on 12/2/21**

| #1 | TS=("Multiple sclerosis" OR "myelin oligodendrocyte glycoprotein antibody associated disease$" OR NMOSD OR MOGAD OR "NMO Spectrum Disorder$" OR "Neuromyelitis Optica Spectrum Disorder$" OR "Devic* Neuromyelitis Optica$" OR "Devic* Disease$" OR "Devic* Syndrome$" OR "Neuromyelitis optica" OR RRMS OR PPMS OR "relapsing remitting MS" OR "remitting relapsing MS" OR "primary progressive MS" OR "acute relapsing MS" OR "Disseminated Sclerosis" or "Acute Fulminating MS") |
| --- | --- |
| #2 | TS=(Coronavirus OR pneumonia virus* OR cov) AND TS=(outbreak OR wuhan OR novel OR 19 OR 2019 OR epidem* OR epidemy OR epidemic* OR pandem* OR new) OR TS=(coronavirus* OR "corona virus*" OR ncov OR 2019ncov OR covid* OR "sars cov 2" OR sars2 OR sarscov2 OR sarscov-2 OR "ncov 2019" OR "sars coronavirus 2" OR "sars corona virus 2" OR "severe acute respiratory syndrome cov 2" OR "severe acute respiratory syndrome cov2" OR "severe acute respiratory syndrome cov*") |
| #3 | TS=(diagnos* OR relaps* OR exacerbat* OR manifest* OR recurrence$ OR worsen* OR "flare up$" OR "flaring up" OR flareup$ OR flare-up$ OR "symptom flare$" OR "symptom* increase$" OR "symptom magnification$" OR "symptom exaggeration$" OR "disease burden") |
| #4 | Limit publication date to to 2019-01-01 to 2022-01-01 |
